# Occlusion-Based Explanations in Deep Recurrent Models for Biomedical Signals

**DOI:** 10.3390/e23081064

**Published:** 2021-08-17

**Authors:** Michele Resta, Anna Monreale, Davide Bacciu

**Affiliations:** 1 Computer Science Department, University of Pisa, 56127 Pisa, Italy; bacciu@di.unipi.it; 2 KDDLab, Computer Science Department, University of Pisa, 56127 Pisa, Italy; anna.monreale@unipi.it

**Keywords:** interpretability, occlusion, recurrent networks, biomedical signals

## Abstract

The biomedical field is characterized by an ever-increasing production of sequential data, which often come in the form of biosignals capturing the time-evolution of physiological processes, such as blood pressure and brain activity. This has motivated a large body of research dealing with the development of machine learning techniques for the predictive analysis of such biosignals. Unfortunately, in high-stakes decision making, such as clinical diagnosis, the opacity of machine learning models becomes a crucial aspect to be addressed in order to increase the trust and adoption of AI technology. In this paper, we propose a model agnostic explanation method, based on occlusion, that enables the learning of the input’s influence on the model predictions. We specifically target problems involving the predictive analysis of time-series data and the models that are typically used to deal with data of such nature, i.e., recurrent neural networks. Our approach is able to provide two different kinds of explanations: one suitable for technical experts, who need to verify the quality and correctness of machine learning models, and one suited to physicians, who need to understand the rationale underlying the prediction to make aware decisions. A wide experimentation on different physiological data demonstrates the effectiveness of our approach both in classification and regression tasks.

## 1. Introduction

The increasing amount of data generated in each field of human activity, paired with the increasing availability of computing power, has contributed to the success of Machine Learning models. Deep Learning systems, in particular, have gained a lot of traction in the last 10 years thanks to their ability to build an internal representation at different levels of abstraction [1]. This feature, along with the high accuracy exhibited in a variety of different settings, largely contributed to their adoption.

In the biomedical domain, Deep Learning has been applied to a variety of different tasks. One area of active study is related to the processing of one-dimensional physiological signals, with the majority of contributions focusing on classification [2]. Applying machine learning techniques also in a regression setting is of particular interest in this field as it enables new non-invasive monitoring techniques for several physiological signals, such as arterial blood pressure (ABP). Research has been conducted to estimate APB from several other signals, such as Photoplethysmogram (PPG) [3] or Electrocardiogram (ECG) and heart rate [4].

Given their inherently black-box nature, Deep Learning systems pose key challenges in the biomedical field where transparency is a critical feature. To trust a model, a clinician needs to know *why* such model is generating the predictions he/she is seeing. The same is true for patients who have the right to know the reasons behind a decision or a diagnosis. This need for transparency and interpretability has fostered a research effort targeting the development of models and techniques to gain insight and possibly an understanding of the models’ predictions and their inner workings [5,6,7,8,9]. This large body of research literature, however, is mostly limited to models for static data types, including flat vectorial information or images. On the other hand, a large share of the data produced in the life sciences is of sequential nature, these being time-series of physiological measurements, such as blood pressure, heart rate, electrodermal activity, or genomic/proteomic chunks.

In this paper, we attempt to fill this gap by specifically targeting the explainability within the context of recurrent neural networks for biomedical signals represented as time-varying sequential data. Within this context, we propose a model agnostic technique (based on systematic occlusion study) to gain granular knowledge about input influence on the predictions of the model. We do so while providing a multi-faceted access to interpretability, considering both the point of view of the machine learning practitioner and the life-science expert, providing targeted explanations for the two reference populations. Our approach is especially designed for explaining the black-box regressors, but we also discuss how it can be adapted for explaining the classification of time series. We evaluated our method on three different datasets of physiological signals in both regression and classification tasks. The remaining of the paper is organized as follows. Section 2 discusses related works. Section 3 formalizes the problem faced and introduces basic concepts for the explanation method, which is described in Section 4. Experimental results are presented in Section 5 and  Section 6. Section 7 concludes the paper.

## 2. Related Works

Interpretability is a multi-faceted problem, and even though it has recently received much attention and different explanation approaches have been proposed [5,6,7,8], a singular shared formalization is still lacking [10]. Explanation methods can be categorized as model-agnostic or model-specific, depending on whether they take into consideration the knowledge of the internal structure of the black box or not.

According to the type of explanations provided by a methodology, we can further differentiate between local and global methods: the former ones generate explanations for specific data instances, while the latter for the logic of the black box as a whole [8].

Some local explanation methods leverage gradient-based methods in order to identify relevant features [11,12,13]. Layer-wise relevance propagation (LRP) [14], instead, makes explicit use of the network activations. The core idea is to find a relevance score for each input dimension starting from the magnitude of the output. The backpropagation procedure implemented by LRP is subject to a conservation property: the relevance score received by a neuron must be redistributed to the lower layers in the same amount. Several different rules were proposed to favour a positive contribution or to generate sparser saliency heatmaps. The Integrated Gradients method [15] combines the sensitivity property of LRP and guarantees the implementation invariance property: if two models are functionally equivalent then the attributions are identical for both. LIME [16] and SHAP [7] are two well-known local methods. The first one generates a simpler interpretable model that approximates the behaviour of the black box in the specific neighbourhood of the instance to be explained. SHAP [7] is a framework that defines a class of additive feature attribution methods and uses a game theoretic approach to assign an importance score to each feature involved in a particular prediction. LRP [14], DeepLIFT [13], and LIME [16] can be considered particular instances of this class of methods.

For models that use attention [17], it is possible to inspect and visualize the learned weights to gain insights on the assigned importance for a given input instance. This approach has been widely applied for model inspection on different types of data and fields, including the biomedical one. RETAIN [18] is an RNN-based model for the analysis of electronic health record (EHR) data. It employs an attention mechanism that allegedly mimics the modus operandi of a clinician: higher weight is given to recent clinical events in the EHR to generate a prediction. The timeline [9] predicts the next category of a medical visit given past EHRs. First, it calculates a low-dimensional embedding of the medical codes of a given EHR; then, a self-attention mechanism generates a context vector. This context vector is then multiplied by a coefficient obtained from a specifically designed function, which takes into account the specific diseases and the time interval. The resulting visit representation vector is the input of a classifier. Given the presence of the multiplier coefficients, it is possible to know how much a specific event contributed to the prediction of the next visit. In [19], the authors show that time steps closer to therapy was associated with higher attention weights and were more influential on the prediction. An adaptation of Class Activation Mapping [15] to 1D time series is described in [20] and applied to Atrial Fibrillation Classification.

Models can also be explained by generating or querying prototypical instances that are representatives of specific output classes. PatchX [21] uses patches to segment the input time series. It extracts local patterns and classifies each of them according to the occurrence of the pattern in a given class. The classification outcome for a complete time series depends on the classes associated with each pattern within it. Other prototype-based approaches leverage the latent representation learned by autoencoders to generate explanations as in [22,23], but in this case, there is a trade-off between prototype quality and classification accuracy.

In [24], the explanations and prototypes are extracted using an information theoretic approach. The authors take the user’s understanding into consideration, which is modelled as a function of the input x of the systems: $u\left(\cdot \right):R^{n}\to R:x\mapsto u:=u\left(x\right)$ and can be seen as a summary of that specific input. Similarly, an explanation e:=e(x) is a quantity presented to the users to help in the understanding of a specific prediction $\hat{y}$. By considering the data points as independent and identically distributed (i.i.d.) realizations of a random variable, the conditional Mutual Information $I(e;\hat{y}|u)$ represents the amount by which the explanation reduces the uncertainty about the prediction.

Our brief literature survey highlights that most of the interpretability methods are tailored to specific settings and sometimes learning architectures. Model agnostic techniques exist but are applied almost exclusively to classification problems and rarely to regression. Additionally, the availability of approaches for sequential data is substantially lower and limited to classification tasks and, sometimes, to forecasting scenarios [8]. The sequence generation setting is left with few approaches, such as [20], adapted from different tasks that need access to the internals of the models. The method proposed in this paper attempts to overcome such limitations by introducing a model agnostic method that can generate explanations in sequential data processing tasks comprising both regression and classification tasks.

## 3. Problem Statement

In this paper, we address the problem of explaining the behaviour of a black box model *b* in the prediction of a time series *y* given a multivariate time series $X=\{x_{1},x_{2},\cdots ,x_{n}\}$.

A prediction dataset X,Y, thus, consists of a set $X=\left\{X_{1},X_{2},\cdots ,X_{s}\right\}\in R^{s\times h\times n}$ of multivariate time series, where we have a target univariate time series $Y\in R^{s\times h}$ assigned to each multivariate one. A multivariate time series *X* consists of *n* univariate time series, each one with *h* time points $x=\left\{t_{1},t_{2},\cdots ,t_{h}\right\}\in R^{h}$. For instance, a single univariate time series can model an ECG signal. In the following, we also use the term *signal* to indicate a single univariate time series. We name a local subsection of a signal a *sub-signal*.

**Definition** **1** (Sub-signal). *Given a signal $x\in R^{h}$, a sub-signal $x^{\prime }$ of x with length w<h is a sequence of w contiguous data points of x, i.e., $x^{\prime }=\left\{t_{p},\cdots ,t_{p+w-1}\right\}$ for 1≤p≤h−w+1.*

Given a black box, time series predictor *b* and a multivariate time series *X* s.t. b(X)=y, our aim is to provide an explanation for the decision b(X)=y. We use the notation b(X)=Y as a shorthand for {b(X)|X∈X}=Y. We assume that *b* can be queried at will.

## 4. The mime Method

We approach the above explanation problem proposing mime (Masking Inputs for Model agnostic local Explanation), a method aiming at understanding why a recurrent neural network outputs a specific prediction and how it reacts to engineered changes in the input signal by using a methodology rooted on occlusions. By occlusion, we denote the alteration of a part of the input signals with a given value. This kind of technique has been applied to analyse the robustness of image classifiers, where important features of the image are masked to observe changes in the predicted class [25].

mime produces an explanation targeted at two different types of users: physicians and technical experts. Physicians receive information about the importance of a particular input signal for the final prediction and information about some particular parts of the input signals influencing the prediction. This information is supported by visualizations. Technical experts instead can use mime to analyse the robustness of the prediction model against some input perturbation.

The different components of our explanation are obtained by using the occlusion mechanism. The occlusion approach proposed in this work does not require prior knowledge concerning the data structure and distribution, and it only requires having access to input signals and model predictions. For each of the sequential input time series of the model, we generate an occluded version by substituting the original signal values with a user-defined value. The alteration can be chosen to last for the whole signal or for a fixed time-span. In the latter case, a windowed approach is employed to systematically analyse the effect of occluding different parts of each input signal.

**Figure 1:**
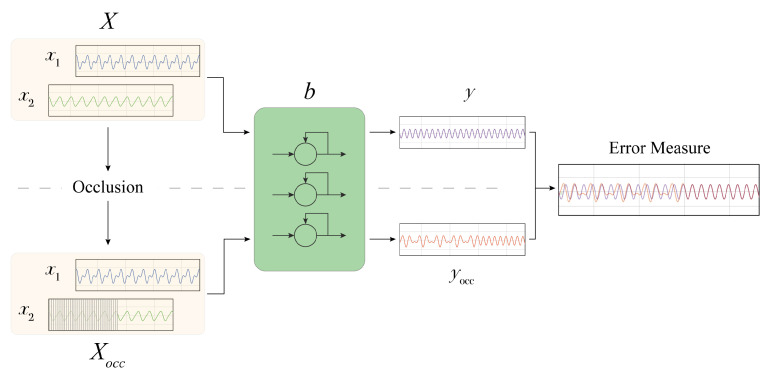
MIME overview. The original input *X* is occluded, generating $X_{occ}$. A black box model *b* generates predictions *y* and $y_{occ}$ using both altered and unaltered inputs. The two predictions are compared using an error measure (e.g., MAE).

In the following (Figure 1), we provide a step-by-step description of the proposed methodology, which includes: *(i)* The determination of the importance of each input signal; *(ii)* Analysis of the impact of the input signals perturbation; *(iii)* The extraction of the most influential *sub-signals*.

### 4.1. Occlusion Approach

Let $X\in R^{s\times h\times n}$ be a tensor representing samples of multivariate time series composed of *n* signals of the length *h*. Each signal *x* is represented by a vector $x\in R^{h}$. We use 1 and 0 to denote vectors whose components are, respectively, all ones and all zeroes. The altered signal $\hat{x}$ is obtained according to the type of modification required. In the case of a full length occlusion, we have: $\hat{x}=o_{v}1$ with $o_{v}$ being the occluding value and $1\in R^{h}$.

In order to modify *x* with a localized alteration of duration *d* starting after *p* timesteps, we define two binary masking vectors $m_{1}$ and $m_{2}$ as:

(1) $$\begin{array}{c} m_{1}=\left(1_{a},0,1_{b}\right)\;\\ m_{2}=\neg m_{1}\\ 1_{a}\in R^{p},\;0\in R^{d},\;1_{b}\in R^{h-(p+d)} \end{array}$$

where ¬ is bit-wise negation. By means of the above masks, we get the $\hat{x}$ vector as:

(2) $$\hat{x}=\left(x⊙m_{1}\right)+o_{v}m_{2}$$

The localized alteration provides the basic elements to define an occlusion approach based on a window *w* covering a specific temporal range.

Given a multivariate time series *X*, we define an occlusion window *w* with a duration of *d* timesteps, and we derive the number of possible segments of a signal that we can occlude, i.e., $q=\frac{h}{d}+c$, where c=0 if the signal duration is divisible by *d*; otherwise, c=1.

Signal occlusion is performed on each segment *i* with i∈[1⋯q]. For each *x*, we alter only a single segment per time. The alteration can be performed on any of the signals $x_{j}\in X$ with j∈[1,⋯n], one at time or by considering any subset of signals in *X*. Algorithm 1 reports the occlusion procedure for a single signal.

By generating the occlusions, we collect the model outputs for both the unaltered input samples Y=b(X) and under the occluded inputs $\hat{X}$, i.e., $Y_{occ}=b\left(\hat{X}\right)$. Then, we consider the discrepancies between the two output signals measured in terms of mean absolute error (MAE) between *Y* and $Y_{occ}$. Thus, higher values of MAE denote higher importance of the occluded signal parts. This approach allows us to investigate several aspects of the models trained for different tasks in the biomedical domain and to extract and analyse explanations. We discuss these aspects in the following sections.
**Algorithm 1** Occlusion.1:**procedure**occlude($x,w_{size},w_{idx}$)2:    len←length(x)3:    $x_{occ}\leftarrow$copy(x)4:    $o_{v}\leftarrow 0$        ▹ user-defined occlusion value.5:    $start\leftarrow w_{size}\cdot w_{idx}$6:    $end\leftarrow start+w_{size}$7:    **if** end>len **then**8:        end←len9:    **end if**10:    **for** i←start,end **do**11:        $x_{occ}\left[i\right]\leftarrow o_{v}$        ▹ window occlusion12:    **end for**13:    **return** $x_{occ}$14:**end procedure**

### 4.2. Input Signal Importance

The first step of mime aims at determining the importance of each input signal for the prediction task. A large number of approaches have been developed to investigate feature importance in machine learning models for interpretability purposes. Most of them are specifically designed to deal with classification tasks, while others (such as SHAP [7]) rely on assumptions that are not always valid, such as the independence of the input features. As an example, in our setting, two input signals, such as cardiac and respiratory data, cannot be considered independent.

In our approach, for each input signal x∈X, we evaluate the importance of *x* by applying the black box *b* on both the data with the entire signal *x* occluded and the original data without any occlusion. The MAE resulting from the comparison of the two predictions quantifies the importance of the signal *x*. Occluding the entire signal means considering a window with a size equal to the signal length, i.e., setting $w_{size}=h$ and $w_{idx}=1$ in Algorithm 1.

### 4.3. Estimating Duration of Induced Perturbation

Occluding parts of the input signals results in an alteration in the network outputs. The predicted signals under input occlusion manifest a perturbation that, as the empirical analysis will show, is clearly visible when plotting the two generated outputs. Following up on this intuition, we developed a procedure to quantify the duration of the induced alteration.

The rationale of our duration estimation procedure follows the approach discussed previously for the signal importance assessment. For any segment occluded in the input signals, we quantify the deviation of the occluded prediction from the unaltered one by computing their MAE over a window of *d* timesteps. In particular, given the two predicted signals *y* and $y_{occ}$, we apply the procedure described in Algorithm 2. First, we segment the two signals in $q=\frac{h}{d}+c$ sub-signals (with c=0 if *h* is divisible by *d*, c=1 otherwise), obtaining two lists of sub-signals *s* and $s_{occ}$, respectively, (lines 4–5, Algorithm 2). Then, we compute the MAE for any pair of aligned sub-signals, i.e., $\forall i\in \left[1\ldots v\right].\;MAE\left(s^{i},s_{occ}^{i}\right)$ (lines 6–9). Perturbation duration is quantified by counting the number of sub-signals for which the MAE is above a threshold $T_{MAE}$ (lines 10–15), whose value is application-dependent.
**Algorithm 2** Perturbation duration.1:**procedure**PertDuration($y,y_{occ},T_{MAE}$)2:    $w_{size}\leftarrow d$        ▹ user-defined size3:    $mae_{l}\leftarrow$ empty list4:    s←Segment($y,w_{size}$)5:    $s_{occ}\leftarrow$Segment($y_{occ},w_{size}$)6:    **for all** $s^{i}\in s$ **do**7:        ϵ←mae($s^{i},s_{occ}^{i}$)8:        Append ϵ to $mae_{l}$9:    **end for**10:    $w_{c}\leftarrow 0$11:    **for all** $\epsilon \in mae_{l}$ **do**12:        **if** $\epsilon >T_{MAE}$ **then**13:           $w_{c}\leftarrow w_{c}+1$14:        **end if**15:    **end for**16:    **return** $w_{c}$        ▹ n. windows with MAE > $T_{MAE}$17:**end procedure**

### 4.4. Determining Influential Sub-Signals

The windowed occlusion procedure can also serve to identify the most relevant or influential input sub-signals for the model. This is, again, obtained by contrasting original predictions with the model outputs under occlusion, measuring the mean discrepancy between the two. Algorithm 3 describes the details of our approach. In particular, it computes, for each input signal x∈X, the importance of each sub-signal of *x*. To this end, the input signal *x* is segmented in *q* sub-signals $s^{1},\ldots ,s^{q}$ (line 4), and for each $s^{i}$, an occluded version of the signal *x* is computed (line 6). Then, the importance of the sub-signal $s^{i}$ is measured by computing the derived MAE comparing the model prediction *y* on the unaltered signal and $y_{occ}$ on the occluded signal (lines 8–10). Once the MAE is computed for each sub-signal, the algorithm produces a heatmap that provides a visual inspection that highlights the importance (measured by MAE) of each sub-signal (see Figure 3 as an example). Finally, the method extracts the top-*k* sub-signals with the highest MAE.

Next, the top-*k* sub-signals of each signal are used to provide the physicians with a set of important sub-signals of each category of the input signal. To this end, given the whole set of multivariate time series X, mime selects from each multivariate X∈X the single univariate signals $x_{j}$ and extracts the top-*k* sub-signals with the highest MAE, which we denote by $TK_{j}^{X}$ (Algorithm 3).

Finally, mime derives the set *I* by computing the union of these top sub-signals obtained for each of the *j*-th signals, i.e., $I=\cup_{X\in X}TK_{j}^{X}$. Finally, it extracts the most important ones from such set, again relying on the MAE values.
**Algorithm 3** Top-K influential Sub-signals. 1:**procedure**TopkSub-Signals($X,x,model,w_{size},k$) 2:    mae_signal← empty list 3:    subsignals← empty list 4:    s←Segment($x,w_{size}$) 5:    **for** i←1,|s| **do** 6:        $x_{occ}\leftarrow$occlude($x,w_{size},i$) 7:        $X_{occ}\leftarrow \left(X\setminus \left\{x\right\}\right)\cup \left\{x_{occ}\right\}$ 8:        $y_{occ}\leftarrow$predict($X_{occ},model$) 9:        y←predict(X,model)10:        ϵ←mae($y,y_{occ}$)11:        Append $\left(\epsilon ,s^{i}\right)$ to mae_signal12:    **end for**13:    mae_signal←ReverseSort(mae_signal)14:    **for** j←1,k **do**15:        Append mae_signal.get(i)[1] to subsignals16:    **end for**17:    **return** subsignals18:**end procedure**

### 4.5. Self Organizing Maps Clustering of Influential Sub-Signals

The set *I* of influential sub-signals, extracted using the procedure described in the previous section, is then used as input for a Self Organizing Map (SOM) [26]. SOMs are the most popular family of neural-based approaches to topographic mapping. They leverage soft-competition among neighbouring neurons arranged on low-dimensional lattices to enforce the principle of topographic organization. Soft-competition ensures that nearby neurons respond to similar inputs, while lattice organization provides a straightforward means to visualize high-dimensional data onto simple topographic structures. Thanks to these characteristics, they have found wide application as an effective computational methodology for adaptive data exploration [27].

In this work, SOMs are used as a visualization tool targeted to domain experts. Thanks to the SOM ability to cluster signals by their morphological similarity and mapping them to a specific neuron, or more generally, to a neighbourhood of neurons, it is possible to obtain a synthetic and organized view of those signals. Exploiting the ability to project auxiliary information such as the MAE linked to each sub-signal in *I*, it is possible to identify prototypical portions of signals associated with the highest error. This process allows us to provide physicians with an intuitive tool to identify and visualize the “critical” parts of the signals. For the sake of our analysis, we can use all sub-signals in the original set *I*, or alternatively, we can operate on a subset *G*, obtained by selecting the most informative *n* (i.e., the ones with the highest error) elements from *I*.

After the training phase, we query the SOM to obtain the best matching unit (BMU) $\forall s^{i}\in I$ and link the BMU to the MAE associated with $s^{i}$. The set of all sub-signals mapped to the BMU with coordinates (u,v) is denoted by $S_{u,v}=\left[s^{1},s^{2},\cdots ,s^{z}\right]$. We build a matrix $E\in R^{u\times v}$ with the same dimensions of the map where each element E[u,v] is:

$$E\left[u,v\right]=\frac{\sum_{z=1}^{|S_{u,v}|}MAE\left(s^{z}\right)}{|S_{u,v}|}\;s^{z}\in S_{u,v}$$

By projecting the matrix *E* on top of the original SOM map, we can easily identify neurons that react to sub-signals with a larger MAE thanks to colour intensity. Sub-signals associated with each BMU can be plotted in isolation or can be linked back to the original input signals they were extracted from, highlighting critical portions of the original time series.

### 4.6. Explaining Time Series Classification

As described above, mime is designed to explain regression tasks. However, it can be easily adapted for providing explanations in time series classification tasks. In this case, each multivariate time series is assigned to a label, i.e., the target $Y\in R^{s}$. In order to adapt our approach to these tasks, we propose determining the signal influence (Section 4.2) and the most influential sub-signals (Section 4.4) by computing the MAE discrepancy between the model losses for the occluded and original signals rather than between the model outputs. Moreover, when selecting the influential sub-signals, we will look into those that lead the model to change its classification prediction. That is why we adapt the approach to return the sub-signals that have the highest MAE and $y_{occ}\neq y$. Clearly, since the prediction is a class label here and there is no temporal information associated with the target, we cannot provide the analysis on the impact of the perturbation in terms of duration of the induced alteration.

All in all, the approach needs to be customized based on whether the predictive task is a regression or a classification problem. In a regression setting, the only actionable choice is the selection of the discrepancy function. For the sake of this work, we measure occluded-unoccluded output discrepancy using MAE. For classification problems, in Algorithm 3, we need to compute the MAE discrepancy between the model losses for the occluded and original inputs rather than between the model outputs. Moreover, when selecting the influential sub-signals, we are interested in those that cause the system to change its classification prediction. For this reason, we append the tuple $\left(\epsilon ,s^{i}\right)$ to the list of the candidate-important sub-signals (line 11) if and only if $y_{occ}\neq y$.

## 5. Experimental Setup

We tested the approach on both classification and regression tasks using several models trained on three different datasets of physiological signals. In this section, we detail the dataset employed and the models used in the experimental assessment.

### 5.1. Datasets

The first set of signals is from the *Cuff-Less Blood Pressure Estimation Data Set* (CBPEDS) [28] available in the UCI ML repository [29]. CBPEDS contains a subset of the physiological signals available in MIMIC II Waveform Database [30] that are useful to create systems for non-invasive blood pressure estimation. MIMIC II is part of PhysioBank [31]. Three different types of synchronized patients recordings are available: electrocardiograms (ECG), photoplethysmograph from fingertip (PPG) and invasive arterial blood pressure (ABP).

The second dataset is the *Combined measurement of ECG, Breathing and Seismocardiograms Database* [32] (CEBSDB), which was constructed to compare RR time series of ECG and seismocardiograms (SCG). Signals were collected by asking 20 presumed healthy volunteers to be very still in a supine position on a comfortable conventional single bed and awake. The subjects were monitored in a basal state for 5 min, for 50 min while listening to classical music, and for another 5 min after the music ended. From this dataset, we used all the available recordings with exception of “ECG lead I”.

To test the approach on a classification task, we used a dataset obtained from the *PTB Diagnostic ECG Database* (PTBDB) [33]. A set of ECG beats were extracted from the original 549 full-length recordings. The nine diagnostic classes (eight for unhealthy heart conditions, one for healthy) in the original dataset were condensed into two classes: one for healthy beats and the other for pathologic conditions. We remand to [34] for details regarding preprocessing and beat extraction.

A summary of the main characteristics of these datasets is available in Table 1. Details on datasets preprocessing are reported in Appendix A.

### 5.2. Models

A total of 9 different models were trained, 3 for each dataset. Given the temporal nature of the physiological signals under analysis, Recurrent Neural Networks models were used. We trained 2 RNN models together with a third non-recurrent one to be used as a baseline competitor. Models were implemented using Keras [35] with Tensorflow 2.0 [36] backend.

Using signals from CBPEDS, we trained the models for the task of estimating the full-length ABP signal using ECG and PPG signals as inputs. On this regression setting, we selected the following models:

- a convolutional autoencoder (AUT) [37] composed of a total of 26 layers: 15 for the encoder and 10 for the decoder;
- a Gated Recurrent Units network (GRU) [38] composed of 5 layers, with a single output;
- a convolutional GRU (CNN-GRU) [39] network of 5 layers and a single output.

A similar regression task was designed with signals from CEBSDB. With the ECG and Breathing signals as input, we predict the whole SCG signal. Given the similarity of the two regression tasks, the six models share most of the architectural choices. Some hyperparameters were tuned to adapt the models to the specific task (details in Appendix B).

We also trained 3 additional models in a binary classification setting using the ECG signals from the PTBDB dataset:

- a fully connected feed forward neural network (MLP) [40] composed of 5 layers;
- a Gated Recurrent Units network (GRU) [38] composed of 4 layers, with a single output;
- a convolutional GRU (CNN-GRU) [39] network of 4 layers and a single output.

Differently from the regression setting, in this case, we used a fully connected network (MLP) as a baseline. This choice is motivated by the fact that it exhibited predictive performances comparable with those of the recurrent models. For all models, the dataset was split into 3 parts: 70% of the data has been used for the training, 10% for validation and 20% for the test set. Networks trained on CEBSDB and CBPEDS used the Mean Absolute Error (MAE) as the loss function, while Binary Cross Entropy was used for models trained on PTBDB.

In the following, we denote models trained on each dataset with the subscripts α, β and δ, for the CBPEDS, CEBSDB and PTBDB, respectively. Table 2 summarizes model performances in the unoccluded case.

## 6. Experiments

In the following sections, we describe the results of the experiments performed using the mime explainer. First, we report results for signal importance assessment using both whole length occlusion and the windowed approach. Next, we describe the analysis pertaining to the duration of induced perturbations. Following, we detail experiments to extract the most influential sub-signals and the associated SOM-based visualizations. Lastly, we provide examples of the *Signal Occlusion Contribution Visualization* targeting the clinical experts.

### 6.1. Signal Importance

Experiments to quantify signal importance for models trained on regression tasks (CBPEDS and CEBSDB) were performed by occluding segments of the input signal with zero values or with the mean value of the dataset for the whole duration. The effects have been evaluated on the validation and test sets from both datasets.

Table 3 reports the results for models trained on the CBPEDS dataset. We include the MAE with the unaltered input as a reference. Different models with different inductive biases learn different representations, and in doing so, they assign different levels of importance to the input signals. The table highlights (in boldtype) that the ${\mathrm{GRU}}_{\alpha }$ model relies more on the PPG signal, as occluding it results in a larger MAE. We have similar results for the ${\mathrm{AUT}}_{\alpha }$ model, while the ${\mathrm{CNN}-\mathrm{GRU}}_{\alpha }$ model, instead, has a larger MAE when the ECG signal is occluded. The type of occlusion seems to play a secondary role, probably related to samples distribution, as results on the validation set indicates. The most important input signals remain the same for all three models, with MAE score variations according to the occlusion type.

**Figure 2:**
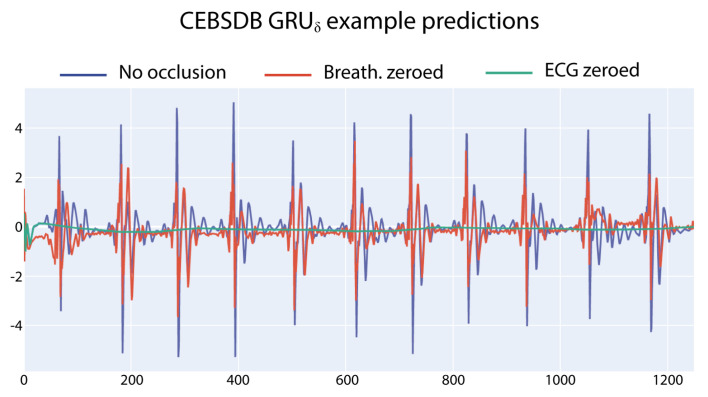
${\mathrm{GRU}}_{\beta }$ predictions for the SCG with occluded input signals. With ECG occluded, the output prediction is a signal oscillating around zero values.

For the CEBSDB dataset, the signal importance assessment in Table 4 reveals a strong reliance of all the three networks on the ECG input signal to correctly generate the SCG output signal. This behaviour is evident when analysing the errors in Table 4: the MAE associated with ECG occlusion is always higher, with the only exception of the ${\mathrm{GRU}}_{\beta }$ model on the test set. The occlusion value has a strong impact on the autoencoder model, while the effects are of smaller magnitude than for the other models. Figure 2 shows a graphical example of the different outputs of the ${\mathrm{GRU}}_{\beta }$ model (trained to predict the SCG ) when different input signals are occluded.

### 6.2. Windowed Occlusion

In this section, we report the results obtained by occluding the input signals with zero values for a fixed window of time for all window indexes. This approach has been applied in both classification and regression models. In the former case, we report the average MAE error obtained across all the windows, and in the latter case, the mean accuracy obtained by considering the occluded prediction.

Table 5 shows the results on CBPEDS datasets. In general, larger mean MAE values are associated with the occlusion of the most meaningful sub-signals, and the error increases with the window size. In predicting the arterial blood pressure, the ${\mathrm{GRU}}_{\alpha }$ model exhibits larger errors when ECG is occluded. The autoencoder is the worst performer of the three models when the PPG signal is occluded, while the ${\mathrm{CNN}-\mathrm{GRU}}_{\alpha }$ model is the most robust among the tested networks.

Table 6 reports results on the CEBSDB dataset. In this regression task, the ${\mathrm{AUT}}_{\beta }$ model is the most susceptible model when the ECG signal is occluded, while the ${\mathrm{CNN}-\mathrm{GRU}}_{\beta }$, as in the ABP estimation task, is less influenced by the occlusion. The ${\mathrm{GRU}}_{\beta }$ model confirms its larger reliance on the breathing signal compared to the other networks, as the associated MAE shows.

The accuracy results obtained with the three different models for the classification task on PTBDB are reported in Table 7. Here, the ECG is the only input signal, and we experimented with different occlusion values. Several window sizes were tested with duration of 25, 50, 75, 100 and 125 time steps. The choice of the occlusion value (zero or mean signal value on the dataset) has a negligible impact on the accuracy (from 1% to 4%). Interestingly, all models worsen their prediction when the occlusion is zero, especially at lower window sizes. Increasing the occlusion duration results in a larger accuracy loss for all models, independently from the value used. The feedforward network used as a baseline is the less susceptible model followed by the ${\mathrm{CNN}-\mathrm{GRU}}_{\delta }$ model. The pure GRU model has, in general, the largest accuracy loss.

### 6.3. Induced Perturbation Duration

Table 8 provides the results for the experiments quantifying the duration of the perturbation caused by different occlusion types in CBPEDS. The ${\mathrm{GRU}}_{\alpha }$ model shows the largest sensibility to the alteration of ECG input and takes more timesteps to undo the induced error, which can last up to 250 timesteps (2 s) even for small occlusion durations, confirming the importance of this signal for this specific model. ${\mathrm{CNN}-\mathrm{GRU}}_{\alpha }$ seems to recover faster than the ${\mathrm{GRU}}_{\alpha }$ model. Moreover, the duration of the perturbation is similar for ECG and PPG occlusions. The best model at dealing with the perturbation duration is ${\mathrm{AUT}}_{\alpha }$. Its mean duration is the lowest in the Table, and when ECG is occluded, its effect lasts for zero timesteps. This does not mean, however, that the induced perturbation is zero: it rather indicates that the induced error is less than the chosen tolerance for the MAE.

The results for the CEBSDB task are reported in Table 9: in this setting, the autoencoder needs a larger time to recover from induced perturbation. Occluding the breathing signal causes no perturbation for both ${\mathrm{AUT}}_{\beta }$ and the ${\mathrm{CNN}-\mathrm{GRU}}_{\beta }$, while the effect is low for the ${\mathrm{GRU}}_{\beta }$ model. Compared with the ABP estimation task, perturbation durations are, in general, lower, with the exception of the autoencoder model. This effect may be due to the nature of the predictive task: the SCG signal has higher variability than ABP, which is probably causing models to recover faster from alterations.

### 6.4. Visualizing Sub-Signal Occlusion Effects

The increasing availability of medical datasets motivates the need for tools to make sense of this large amount of information [41]; one of the fastest and most effective ways to convey key aspects of data under analysis is by visualization. The proposed explanation is targeted at experts in the medical domain. By *expert in the medical domain*, we mean a clinician or doctor, that is, a person who has no professional computer science background but rather a medical one.

We get our visualization by overlapping two different kinds of plots. The first one is the plot of the input signal we are considering, which in the case of CBPEDS, is either an ECG curve or a PPG curve. The second one is a windowed heatmap used as a background for the first plot. The heatmap is generated by occluding the signal under analysis for a specific user-defined window of time, with the approach described in Section 4.2.

**Figure 3:**
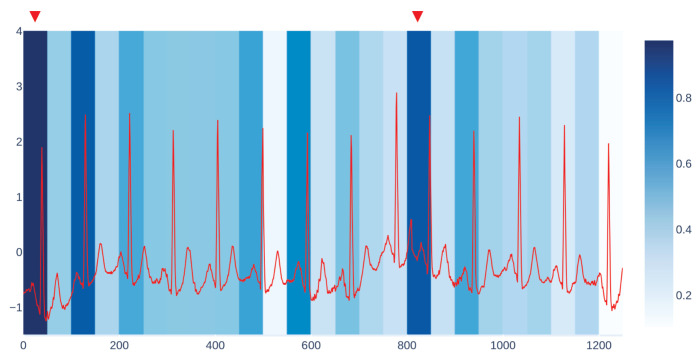
A visualization of sub-signal occlusion contributions for an ECG signal from CBPEDS. The occlusion window size is equal to 50. Red triangles mark the portions of the signal that contribute more to MAE increase.

For each occluded window index, we plot the associated MAE error with a proportionally intense background colour. Figure 3 shows an example of our visualization of the occlusion contribution for an ECG signal from the CBPEDS dataset with a window occlusion size of 50 timesteps. For the ECG signal analysed, it is clear that an occlusion in the first window of the signal results in a higher error. Moreover, the section of the signal around the 800th time step (indicated with a red triangle) is also associated with a high MAE. By observing this visualization, clinicians can get an insight into which portion of the input signals are influential for the output prediction of the model and assess whether the highlighted sub-signals are critical morphological features employed for classical diagnosis methods. Another example of such a visualization for a different window size is reported in Appendix C.

**Figure 4:**
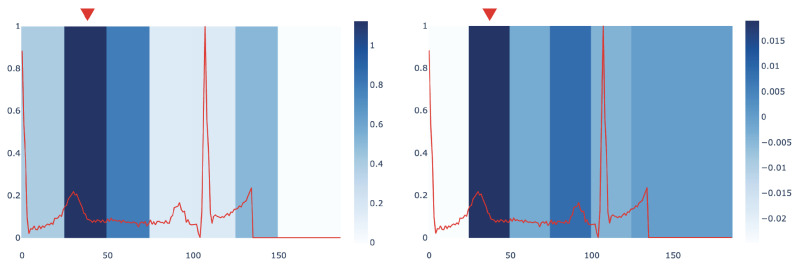
A comparison of importance assigned to sub-signals of an ECG from PTB by mime (**left**) and Integrated Gradients (**right**). The window with the highest score is marked by the red triangle.

In order to assess the interpretation provided by mime, we compared it with the Integrated Gradients (IG) method [15]. Figure 4 compares the most influential sub-signals of an ECG signal identified by mime and IG in PTB dataset. The comparison points out how both methods are concordant in identifying the same important window as the key subsequence in the analysed signal.

We also provide a quantitative evaluation of the concordance between the mime and IG interpretations. To this end, we compute a score measuring when the two methods select the same sub-signal or sub-signals that are temporally close as the most influential ones. Given that, mime returns an importance score for each window of duration *d* over the signal *x* (as explained in Section 4.6). We define an importance score, based on Integrated Gradients, to compare our results with the IG method. In particular, given a window of duration *d*, we calculate this score as the sum of the IG values $IG_{j}$ of each timestep *j*, i.e., $IG_{score}=\sum_{j=1}^{d}IG_{j}$. We assign an index to each window; thus, we can derive from each signal which window index corresponds to the highest importance score in both methods. We name them $index_{IG}$ and $index_{MIME}$. Then, we compute how many windows identified by mime and IG perfectly match or differ by no more than 1 window index, i.e., $|index_{IG}-index_{MIME}|<=1$. A preliminary investigation conducted on ${\mathrm{MLP}}_{\delta }$ found that mime and IG have a concordance score of 68.20% for the signals in PTB dataset. We leave a more in-depth quantitative characterization of the relationships between the two approaches as future work.

### 6.5. Most Influential Sub-Signals

In this section, we describe the SOM-based analysis performed on the most influential samples extracted from the various datasets and according to the different models. The maps were trained using the MiniSOM python library [42]. For each recurrent model, we trained several SOMs using the top 5000 sub-signals extracted from the corresponding training dataset as input. All maps have dimensions (12,17). We used a Gaussian neighbourhood function with σ=2.05 and hexagonal topology. SOMs were trained with a learning rate $l_{r}=0.7$ for a total of $10^{5}$ steps.

**Figure 5:**
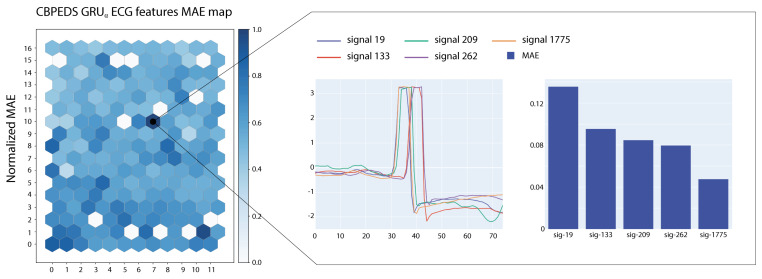
The SOM map with MAE-based colouring for a ${\mathrm{GRU}}_{\alpha }$ model tested on ECG sub-signals.

**Figure 6:**
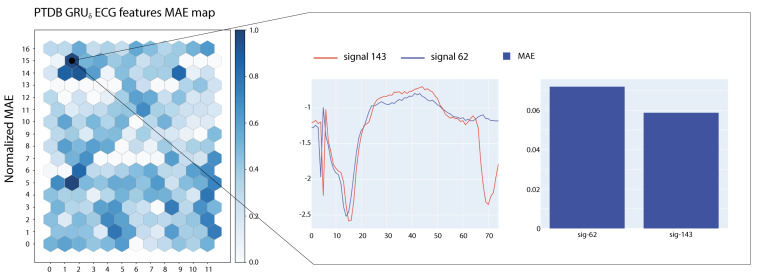
SOM maps with MAE-based colouring for a ${\mathrm{GRU}}_{\delta }$ model tested on ECG sub-signals.

After the training phase, we have tested the SOM with the top 2000 sub-signals extracted from the test portion of each dataset to build the *E* matrix with $E\in R^{12\times 17}$. We normalize *E* to have values in the [0,1] range and project this information on the SOM as a heatmap. Figure 5 and Figure 6 show two examples of visualizations obtained from the SOMs trained on ECG signals. In the figures, we report also a close-up on the prototypical signals associated with the most active neuron in the map. Signals associated with the highlighted neurons show large MAE errors and share morphological characteristics.

Self Organizing Maps obtained from the training dataset can be shared with users of the predictor to help them in assessing the behaviour of the model on novel data. By repeating the most influential sub-signal extraction phase on a production dataset, the SOM can be used to generate an updated visualization. Such visualization will provide a useful global overview of problematic sub-signals of new time series data.

## 7. Conclusions

In this work, we presented an interpretability approach for sequential data based on input occlusion. The approach is model-agnostic and only requires access to model inputs and outputs. Using the proposed methodology, we studied several recurrent neural networks trained on both regression and classification tasks and analysed the importance assigned by the models to each input signal.

Our results highlight how different models rely on different input signals to generate their predictions and show larger errors when that input is occluded. The perturbation induced by occlusion lasts longer when the occluded input are those resulting from the signal importance analysis. In regression tasks, recurrent models are more robust compared to the convolutional autoencoder baselines, with the CNN-GRUs suffering less from input alteration compared to the pure GRU models. The increased robustness is probably due to the convolutional layer providing “look-ahead” capabilities to the recurrent layer.

The simple feedforward network used as a baseline in the classification task is more robust with respect to the two recurrent models. As in the regression setting, the CNN-GRU performed better compared to the vanilla GRU and exhibited a minor loss of classification accuracy.

Moreover, leveraging the occlusion approach, we designed two different visualizations aimed at clinicians. The first one gives a detailed view of the error associated with the occlusion of portions of a single input signal.

The second one is based on Self Organizing Maps and is used to visually inspect and discover critical sub-signals associated with high prediction errors.

Interesting future work directions are the development of a data-driven algorithm to select the optimal occlusion window size and increasing the human–machine interaction degree. The latter would allow the proposed approach to be used in *“what if?"* scenarios, enabling faster comparisons of explanations generated from user-specified parameters.

## Figures and Tables

**Table 1:** Summary of datasets.

	CBPEDS	CEBSDB	PTBDB
# of timeseries	10,158	1512	14,552
# of variables	3	3	1
Length (time points)	1250	1250	187
Sampling freq.	125 Hz	5 kHz	125 Hz
# of classes	-	-	2
# of normal time-series	-	-	4046
# of abnormal time-series	-	-	10,506

**Table 2:** Selected model performances on each dataset.

	CBPEDS (MAE)		CBPEDS (MAE)		PTBDB (Accuracy)	
	**Valid. Set**	**Test Set**	**Valid. Set**	**Test Set**	**Valid. Set**	**Test Set**
${\mathrm{GRU}}_{\alpha }$	14.909	17.636	-	-	-	-
${\mathrm{CNN}-\mathrm{GRU}}_{\alpha }$	14.814	17.826	-	-	-	-
${\mathrm{AUT}}_{\alpha }$	11.744	13.581	-	-	-	-
${\mathrm{GRU}}_{\beta }$	-	-	1.494	1.662	-	-
${\mathrm{CNN}-\mathrm{GRU}}_{\beta }$	-	-	1.451	1.779	-	-
${\mathrm{AUT}}_{\beta }$	-	-	1.315	2.073	-	-
${\mathrm{GRU}}_{\delta }$	-	-	-	-	99.31%	99.18%
${\mathrm{CNN}-\mathrm{GRU}}_{\delta }$	-	-	-	-	96.46%	96.29%
${\mathrm{MLP}}_{\delta }$	-	-	-	-	92.58%	99.18%

**Table 3:** CBPEDS Signal importance results.

Occlusion Type	Validation Set MAE			Test Set MAE		
	${\mathrm{GRU}}_{\alpha }$	${\mathrm{CNN}-\mathrm{GRU}}_{\alpha }$	${\mathrm{AUT}}_{\alpha }$	${\mathrm{GRU}}_{\alpha }$	${\mathrm{CNN}-\mathrm{GRU}}_{\alpha }$	${\mathrm{AUT}}_{\alpha }$
No occlusion	14.90	14.81	11.77	17.63	17.82	13.58
ECG zeroed	17.42	**17.17**	13.70	18.96	19.63	15.68
PPG zeroed	17.32	14.52	**14.21**	**20.01**	18.65	16.18
ECG mean	16.97	17.07	13.74	18.83	**19.71**	15.57
PPG mean	**18.05**	14.57	14.11	19.80	18.23	**16.22**

**Table 4:** CEBSDB signal importance results.

Occlusion Type	Validation Set MAE			Test Set MAE		
	${\mathrm{GRU}}_{\beta }$	${\mathrm{CNN}-\mathrm{GRU}}_{\beta }$	${\mathrm{AUT}}_{\beta }$	${\mathrm{GRU}}_{\beta }$	${\mathrm{CNN}-\mathrm{GRU}}_{\beta }$	${\mathrm{AUT}}_{\beta }$
No occlusion	1.494	1.451	1.315	1.662	1.779	2.073
ECG zeroed	1.862	**1.864**	**2.416**	1.773	**1.786**	2.130
Breathing zeroed	1.532	1.451	1.319	1.897	1.779	1.949
ECG mean	**1.868**	1.863	1.868	1.773	1.786	**2.135**
Breathing mean	1.533	1.426	1.319	**1.899**	1.646	1.954

**Table 5:** CBPEDS mean MAE results for different window sizes. Occlusion with zero values. Lowest values are in bold, highlighting models less affected by occlusion error.

$W_{\mathrm{size}}$	Signal		Test Set $\bar{\mathrm{MAE}}$	
		${\mathrm{GRU}}_{\alpha }$	${\mathrm{CNN}-\mathrm{GRU}}_{\alpha }$	${\mathrm{AUT}}_{\alpha }$
25	ECG	0.619±0.143	0.406±0.076	0.405±0.054
25	PPG	0.522±0.119	0.448±0.094	0.856±0.112
25	Both	0.810±0.170	0.741±0.139	0.959±0.111
75	ECG	1.088±0.254	0.760±0.153	0.810±0.131
75	PPG	1.088±0.239	0.901±0.185	1.232±0.168
75	Both	1.657±0.344	1.328±0.232	1.681±0.224
125	ECG	1.425±0.232	1.080±0.150	1.180±0.140
125	PPG	1.544±0.227	1.299±0.174	1.664±0.190
125	Both	2.507±0.337	1.973±0.213	2.362±0.203

**Table 6:** CEBSDB Mean MAE results for different window sizes. Occlusion with zero values. Lowest values are in bold, highlighting models less affected by occlusion error.

$W_{\mathrm{size}}$	Signal		Test Set $\bar{\mathrm{MAE}}$	
		${\mathrm{GRU}}_{\beta }$	${\mathrm{CNN}-\mathrm{GRU}}_{\beta }$	${\mathrm{AUT}}_{\beta }$
25	ECG	0.061±0.034	0.062±0.025	0.112±0.016
25	Breath	0.068±0.057	0.071±0.032	0.0682±0.010
25	Both	0.093±0.059	0.091±0.036	0.147±0.020
75	ECG	0.129±0.057	0.137±0.056	0.236±0.042
75	Breath	0.127±0.094	0.11±0.053	0.0887±0.017
75	Both	0.183±0.098	0.161±0.067	0.268±0.047
125	ECG	0.194±0.078	0.206±0.073	0.339±0.041
125	Breath	0.187±0.126	0.147±0.068	0.111±0.0183
125	Both	0.263±0.118	0.231±0.082	0.363±0.046

**Table 7:** PTBDB models mean accuracy decrease for different window sizes. Lowest decreases in bold.

			Test Set Accuracy Decrease (%)	
$W_{\mathrm{size}}$	**Occ Value**	${\mathrm{GRU}}_{\delta }$	${\mathrm{CNN}-\mathrm{GRU}}_{\delta }$	${\mathrm{MLP}}_{\delta }$
25	zero	13.18	14.29	**10.86**
25	mean	10.18	12.29	**8.0**
50	zero	20.18	19.29	**16.0**
50	mean	18.18	17.29	**14.0**
75	zero	26.18	23.29	**19.0**
75	mean	26.18	19.19	**17.0**
100	zero	29.18	26.29	**20.0**
100	mean	25.18	26.29	**21.0**
125	zero	28.18	25.29	**21.0**
125	mean	28.18	26.29	**21.0**

**Table 8:** CBPEDS perturbation duration for the different models occluded with zero value. Lowest durations are highlighted in bold.

$W_{\mathrm{size}}$	Signal	Test Set Mean Duration (ts)		
		${\mathrm{GRU}}_{\alpha }$	${\mathrm{CNN}-\mathrm{GRU}}_{\alpha }$	${\mathrm{AUT}}_{\alpha }$
25	ECG	188.00±40.69	138.00±25.61	0.00±0.00
25	PPG	161.50±33.25	136.00±25.57	15.50±12.13
25	Both	206.00±47.05	171.00±35.83	17.00±11.66
75	ECG	91.18±20.90	195.59±43.08	0.00±0.00
75	PPG	244.12±62.15	202.94±45.28	20.59±9.53
75	Both	91.18±20.90	213.24±49.35	23.53±5.88
125	ECG	310.00±68.19	257.50±44.79	0.00±0.00
125	PPG	300.00±64.23	257.50±44.79	25.00±0.00
125	Both	327.50±76.20	280.00±55.68	25.00±0.00

**Table 9:** CEBSDB Perturbation duration for the different models occluded with zero value. Lowest durations are highlighted in bold.

$W_{\mathrm{size}}$	Signal		Test Set Mean Duration (ts)	
		${\mathrm{GRU}}_{\beta }$	${\mathrm{CNN}-\mathrm{GRU}}_{\beta }$	${\mathrm{AUT}}_{\beta }$
25	ECG	0.00±0.00	14.50±12.34	0.00±0.00
25	Breath	0.00±0.00	0.00±0.00	0.00±0.00
25	Both	18.00±12.29	25.50±3.50	1.50±5.94
75	ECG	38.24±17.40	58.82±14.71	91.18±28.36
75	Breath	2.94±11.76	0.00±0.00	0.00±0.00
75	Both	69.12±18.25	75.00±8.57	98.53±26.39
125	ECG	87.50±16.77	117.50±11.46	155.00±18.71
125	Breath	10.00±30.00	0.00±0.00	0.00±0.00
125	Both	122.50±17.50	127.50±7.50	192.50±46.17

**Figure A1:**
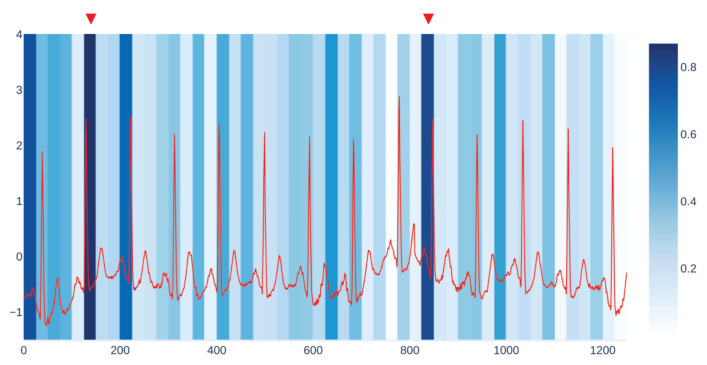
A visualization of sub-signal occlusion contributions for an ECG signal from CBPEDS. The occlusion window size is equal to 25. Red triangles mark the portions of the signal that contribute more to MAE increase.

## Data Availability

Not Applicable.

## Appendix A. Dataset Preprocessing

### Appendix A.1. CBPEDS

The *Cuff-Less Blood Pressure Estimation Data Set* is available from the UCI ML repository. Each entry in the dataset is a multivariate time series containing the following signals:

- ECG signal: electrocardiogram from channel II;
- PPG signal: photoplethysmograph from fingertip;
- ABP signal: invasive arterial blood pressure.

Each signal is collected at a sampling frequency of 125 Hz. All three signals are synchronized for each patient.

Preprocessing steps:

1. Smoothing all signals with a simple averaging filter;
2. Removing signal blocks with irregular and unacceptable human blood pressure values;
3. Removing signal blocks with severe discontinuities, which was not resolved by smoothing filter in step 1;
4. The calculation of PPG signal autocorrelation, which indicates the degree of similarity between successive pulses, and removing blocks with high alteration.
5. Removing samples with irregular readings appearing in at least one of the three signals;
6. Signals duration normalization. Each signal was divided into segments of 10 s.

### Appendix A.2. CEBSDB

The *The combined measurement of ECG, Breathing and Seismocardiograms Database* is available from Physiobank.

Each entry in the dataset is a multivariate time series containing the following signals:

- ECG signal: electrocardiogram from channel I;
- ECG signal: electrocardiogram from channel II;
- Breathing signal: breathing signal from thoracic piezoresistive band;
- SCG signal: seismocardiogram obtained using a triaxial accelerometer.

Each channel was sampled at 5 kHz. All four signals are synchronized for each patient.

Preprocessing steps:

1. removing ECG obtained from channel I from all samples to maintain consistency with the CBPEDS dataset;
2. downsampling all signals to have a duration of 1250 time steps.

## Appendix B. Trained Models

### Appendix B.1. Models for CBPEDS

Adam with a learning rate of $3\times 10^{-3}$ was used as the optimizer, and the batch size was set to 512 for all architectures. The best models were selected according to the best validation score of three training runs.

#### Appendix B.1.1. GRU α Model

The network is composed of five layers:

- **Input:** all the 1250 timesteps of both PPG and ECG are passed as input to this layer;
- **Dense:** a fully connected layer of 256 neurons with linear activation functions;
- **Batch-Norm:** the batch normalization layer. Keras default parameters were used;
- **Recurrent layer:** the recurrent part of the network is based on a GRU of 256 neurons with hyperbolic tangent activations and sigmoid as recurrent activation functions;
- **Output:** the fully-connected layer composed of a single neuron with a linear activation function.

#### Appendix B.1.2. CNN-GRU α Model

The network involves five layers:

- **Input:** ECG and PPG signals are passed in their full length as in the ${\mathrm{GRU}}_{\alpha }$ case;
- **1-D Conv:** one-dimensional convolutional layer composed of a kernel of 6 convolutional filters with a length of 128 time steps. This layer has linear activation function and stride equal to 1;
- **Batch-Norm:** the batch normalization layer. Keras default parameters were used;
- **Recurrent:** the recurrent part of the network is composed of a GRU of 256 neurons with hyperbolic tangent activations and sigmoid as recurrent activation functions;
- **Output:** the fully-connected layer composed of a single neuron with a linear activation function.

#### Appendix B.1.3. AUT α Model

A deep convolutional autoencoder composed of a total of 26 layers. The encoder part of the network comprises 15 layers, and the decoder part counts 10 layers. The encoder is composed of a sequence of four blocks, each composed of a sequence of four layers, except for the last one that is missing the max-pooling layer:

- **1-D Conv:** one-dimensional convolution with a filter bank of 9 filters and ReLU activations. Filter lengths are different in each block. For block one to four, lengths are, respectively, 256, 128, 64 and 16;
- **Batch-Norm:** the batch normalization layer. Keras default parameters were used;
- **Dropout:** random dropout of 15%;
- **Max Pooling:** max pooling is applied with different pool sizes and strides parameters in each block. From block one to three pool sizes are, respectively, 2,5 and 5. The same is valid for stride parameters.

The decoder is composed of three blocks performing transposed convolution to output a signal with the same length of the input. Each block comprises three parts:

- **Up-sampling layer:** repeats input before convolution operation;
- **1-D Conv**: analogous to the ones presented in the encoder but in reverse order. Bank of 9 filters with different lengths, respectively, 32, 64 and 1 for blocks from 1 to 3;
- **Batch-Norm:** identical to the encoder’s one;
- **Dropout:** identical to the encoder’s one.

### Appendix B.2. Models for CEBSDB

Adam with a learning rate of $3\times 10^{-5}$ was used as the optimizer, and the batch size was set to 512 for all architectures. The best models were selected according to the best validation score of three training runs.

#### Appendix B.2.1. GRU β Model

The sequence of the network layer is the same as the GRU model for the CBPEDS, with the difference being that the Dense layer and the GRU layer are composed of 1250 and 64 units, respectively.

#### Appendix B.2.2. CNN-GRU β Model

The architecture of the convolutional GRU is identical to the one used for the CBPEDS. We remand to the previous section for the details.

#### Appendix B.2.3. AUT β Model

The architecture of the autoencoder is identical to the one used for the CBPEDS. We remand to the previous section for the details.

### Appendix B.3. Models for PTBDB

#### Appendix B.3.1. GRU δ Model

This model was trained for 180 epochs using Adam with a learning rate of $3\times 10^{-3}$ and a batch size of 256. It is composed of the following layers:

- **Input:** 187 ECG timesteps;
- **Recurrent layer:** the recurrent part of the network is based on a GRU of 200 neurons with hyperbolic tangent activations and sigmoid as recurrent activation functions;
- **Dense:** the fully connected layer of 100 neurons with ReLU activation functions;
- **Output:** a single neuron with the sigmoid activation function.

#### Appendix B.3.2. CNN-GRU δ Model

This model was trained for 220 epochs using Adam with a learning rate of $3\times 10^{-3}$ and a batch size of 512. It is composed of the following layers:

- **Input:** 187 ECG time steps;
- **1-D Conv:** one-dimentional convolutional layer with a filter bank of 64 kernel of size 30 and stride 1;
- **Recurrent layer:** the recurrent part of the network is composed by a GRU of 32 neurons with hyperbolic tangent activations and sigmoid as recurrent activation functions;
- **Output:** a single neuron with the sigmoid activation function.

#### Appendix B.3.3. MLP δ Model

This model was trained for 100 epochs using Adam with a learning rate of $3\times 10^{-4}$ and a batch size of 512. It is composed of the following layers:

- **Input:** 187 ECG timesteps;
- **Dropout:** random dropout of 10%;
- **Dense:** fully connected layer of 20 units with ReLU activations;
- **Dropout:** random dropout of 20%;
- **Dense:** fully connected layer of 20 units with ReLU activations;
- **Dropout:** random dropout of 20%;
- **Dense:** fully connected layer of 20 units with ReLU activations;
- **Dropout:** random dropout of 30%;
- **Output:** fully-connected layer composed of a single neuron with a sigmoid activation function.

## Appendix C. Visualizing Sub-Signal Occlusion Effects

Here, we show another example of the proposed visualization that highlights sub-signals contributions to the error (Figure A1). The plot shows an ECG signals from CBPEDS occluded with a window size of 25 time steps.
